# Plasticity in voltage-gated ion channels following overwintering in respiratory motoneurons of American bullfrogs

**DOI:** 10.1242/jeb.249687

**Published:** 2025-03-24

**Authors:** Renato Filogonio, Sandy E. Saunders, Michael Gray, Jose A. Viteri, Joseph M. Santin

**Affiliations:** ^1^ Division of Biological Sciences, University of Missouri, Columbia, MO 65201, USA; ^2^ Department of Physical Medicine and Rehabilitation, University of Missouri-Columbia, Columbia, MO 65211, USA

**Keywords:** Breathing control, Hibernation, Hypoglossal, Ion currents, Vagus

## Abstract

Many animals undergo prolonged dormancy periods to survive cold or dry environments. While humans and most laboratory-based mammals experience a loss of neuromuscular function during inactivity, hibernators possess physiological mechanisms to mitigate this loss. The American bullfrog provides an extreme model of this phenomenon, as brainstem circuits that generate breathing are completely inactive during underwater hibernation, during which motoneurons employ various types of synaptic plasticity to ensure adequate respiratory motor output in the spring. In addition to synapses, voltage-gated ion channels may undergo plasticity to boost neuronal output. Therefore, we hypothesized that motoneuron excitability would also be enhanced after hibernation via alterations in voltage-gated ion channels. We used whole-cell patch-clamp electrophysiology to measure membrane excitability and activities of several voltage-gated channels (K^+^, Ca^2+^, Na^+^) from motoneurons that innervate muscles of the buccal pump (hypoglossal) and glottal dilator (vagal). Surprisingly, compared with controls, overwintered hypoglossal motoneurons displayed multiple indices of reduced excitability (hyperpolarized resting membrane potential, lower firing rates, greater lag to first spike). Mechanistically, this occurred via enhanced voltage-gated K^+^ and reduced Ca^2+^ channel activity. In contrast, vagal motoneuron excitability was unaltered, but exhibited altered ion channel profiles which seemed to stabilize neuronal output, involving either reduced Ca^2+^ or K^+^ currents. Therefore, different motoneurons of the same neuromuscular behavior respond differently to overwintering by altering the function of voltage-gated channels. We suggest divergent responses may reflect different energetic demands of these neurons and/or their specific contribution to breathing and other orofacial behaviors.

## Teaser

**Summary:** Hibernating frogs maintain motor function of breathing after spending months underwater not using the relevant muscles and nerves; the molecular components of neurons (ion channels) changes in different ways in different types of neurons to support the activity of this system.

## INTRODUCTION

To survive long cold winters, many vertebrates enter into a dormancy state characterized by an extended period of reduced motor activity and metabolic rates (Withers and Cooper, 2006). In humans and most animal models, disuse of neuromotor processes leads to muscular atrophy and neurological defects (Bonaldo and Sandri, 2013; Clark et al., 2006; Cormery et al., 2005; Deschenes et al., 2002; Seki et al., 2007). However, investigations on hibernators, which experience reduced activity for prolonged periods, have shown that these organisms possess mechanisms that mitigate this loss of neuromuscular function (Hudson and Franklin, 2002). Although there is a wealth of studies exploring the mechanisms preserving muscle function in hibernating species (Cotton, 2016; Dobson, 2004; Ivakine and Cohn, 2014; Ratigan and McKay, 2016; Wickler et al., 1987, 1991; Yacoe, 1983), the question on how neural systems overcome the challenges of reduced activity is still neglected.

An extreme example of neuromuscular inactivity during hibernation occurs in American bullfrogs, *Aquarana catesbeiana* (Shaw, 1802), specifically within the respiratory motor system. Anuran amphibians typically breathe air using lungs. This behavior is driven by a neuromotor circuit in the brainstem, leading to coordinated contraction of various muscles to produce air flow. However, during overwintering, the combined effects of low temperatures and reduced metabolic rates allow the bullfrog and other ranid frogs to sustain gas exchange demands by cutaneous respiration alone, such that frogs may remain underwater for several months without lung ventilation (Santin, 2019; Santin and Hartzler, 2017; Tattersall and Ultsch, 2008). During this period, neural circuits responsible for respiratory motor output stop completely (Santin and Hartzler, 2017). If the motor circuits driving this behavior were to degrade during prolonged inactivity, it would be catastrophic for restarting breathing months later. However, breathing resumes with no apparent functional impairments (Santin and Hartzler, 2016).

Recent work demonstrates that, rather than passively maintaining neuronal function throughout the winter, several types of neuroplasticity are engaged during hibernation to ensure adequate restarting of the respiratory network upon emergence. First, respiratory motoneurons increase excitatory synaptic transmission by enhancing AMPA-glutamate receptors, and thus receive increased excitatory synaptic transmission, further enhancing motor outflow. Second, inhibitory GABAergic transmission is reduced, which serves to increase motor frequency and amplitude at low temperatures (Saunders and Santin, 2024). Finally, as the animal emerges from hibernation, neural activity can resume as a result of several metabolic adjustments in response to hypoxic and hypoglycemic conditions (Amaral-Silva and Santin, 2024; Bueschke et al., 2021a). Thus, these adaptations indicate that several mechanisms ensure the respiratory network restarts to allow lung ventilation upon emergence from hibernation. Therefore, the hibernating bullfrog presents a robust model to understand mechanisms that maintain neuronal function in an extreme model of motor inactivity.

While inactivity is often thought to degrade neuronal function, evidence shows that neurons can monitor their activity levels and fine-tune membrane excitability to stabilize activity during disturbances (i.e. referred to as ‘homeostatic plasticity’; Turrigiano, 2008; Wen and Turrigiano, 2024). In this framework, when neuronal activity falls below a set-point level of activity, alterations in intracellular Ca^2+^ concentration caused by the activity change may serve as a signaling molecule to alter intrinsic excitability by balancing inward and outward currents (Golowasch et al., 1999; LeMasson et al., 1993; Marder and Prinz, 2002; Turrigiano et al., 1994). This may occur by altering voltage-gated ion channel density and the balance of synaptic excitation and inhibition (Desai et al., 1999; Li et al., 1992; Turrigiano, 2011). Therefore, the present study addressed how the hibernation environment influences intrinsic excitability and membrane ionic currents in three motoneuron types of the respiratory network from *A. catesbeiana*.

In anurans, lung inflation occurs via positive pressure elicited through compression of the buccal cavity by activating buccal levator muscles, including those innervated by hypoglossal, facial and trigeminal nerves (Milsom et al., 2022). Specifically, the hypoglossal nerve gives rise to two efferent branches: one innervates the genioglossus muscle, which is responsible for compression of the buccal cavity and originates rostral to obex on the floor of the 4th ventricle; the other innervates the sternohyoid muscle, which is responsible for buccal floor dilation and originates from a more caudal region in the brainstem (Stuesse et al., 1983). Bidirectional lung airflow depends on glottal opening, which is presumably regulated by glottal dilators and constrictors controlled by the vagus nerve (Kogo and Remmers, 1994; Kogo et al., 1994; Milsom et al., 2022; Sakakibara, 1984a). The most caudal branch of the vagal nerve complex innervates laryngeal constrictors and dilators (Sakakibara, 1984b). Recordings of these neurons in a semi-intact brainstem preparation that maintains respiratory bursting showed that ∼75% of these neurons receive respiratory-related synaptic input (Amaral-Silva and Santin, 2022). These cells can be further characterized by their intrinsic membrane properties, where some neurons have the capacity for high-frequency firing and low membrane resistances (‘fast cells’) and others are characterized by comparably lower firing rates and high member resistance (‘slow cells’) (Zubov et al., 2021). Given that previous results at excitatory synapses were consistent with a compensatory response to inactivity in the hibernation environment (Zubov et al., 2022), we hypothesized motoneurons would increase intrinsic excitability to maintain neuronal activity following overwintering. Therefore, we measured firing rates of hypoglossal and vagal motoneurons (fast and slow) from control and cold-acclimated frogs subjected to an overwintering environment without the possibility of lung ventilation. To identify specific compensatory mechanisms that could influence intrinsic excitability, we used patch-clamp electrophysiology to measure passive membrane properties (membrane potential and input resistance), and also several voltage-dependent ionic currents carried by K^+^ (total K^+^ current, delayed-rectifier K^+^ current, A-type K^+^ current), Ca^2+^ (L-type Ca^2+^ current) and Na^+^ (persistent inward Na^+^ current) in vagal and hypoglossal motor neurons from control and overwintered frogs.

## MATERIALS AND METHODS

### Animal acquisition and maintenance

American bullfrogs (*Aquarana catesbeiana*) were acquired from commercial suppliers (Rana Ranch, Twin Falls, ID, USA) and housed at the laboratory of the Division of Biological Sciences at the University of Missouri. Frogs were randomly assigned to control or overwintered groups as soon as they arrived at the laboratory facilities. Animals from both groups were kept within plastic tanks with dechlorinated water constantly bubbled with room air and a 12 h:12 h light:dark cycle. Control frogs were kept in a room at 20°C and fed once a week with pellets provided by the seller. Cold-acclimated frogs were housed in a temperature-controlled room set to 20°C for at least 1 week, after which room temperature was reduced 2°C daily until it reached 4°C. Once the temperature reached 4°C, plastic nets were placed at the water level to impede lung ventilation. Frogs in the overwintering group were not fed and were kept at 4°C for at least 1 month before experiments commenced. All procedures were previously approved by the Animal Care and Use Committee from the University of Missouri (#39264).

### Tissue preparation

Tissues were prepared as previously described (Santin et al., 2017). Frogs were initially anesthetized with isoflurane (1 ml) applied to gauze in a sealed plastic box of ∼1 l in volume. After loss of pedal reflexes (Neto et al., 2024), individuals were euthanized by decapitation. The head was immersed in ice-cold artificial cerebrospinal fluid (aCSF; in mmol l^−1^: 104 NaCl, 4 KCl, 1.4 MgCl_2_, 7.5 d-glucose, 40 NaHCO_3_, 2.5 CaCl_2_ and 1 NaH_2_PO_4_, gassed with 1.5% CO_2_/98.5% O_2_ for pH 7.85), the brainstem–spinal cord was dissected free and the dura removed. For recordings of hypoglossal motoneurons, the brainstem–spinal cord was glued to an agar block and sectioned (300 μm thick) with a vibrating microtome (Technical Products International series 1000, St Louis, MO, USA). For this study, we recorded hypoglossal motoneurons in the rostral part of the motor pool that reside medial to the 4th ventricle, as these neurons innervate the buccal floor compressor muscles (Stuesse et al., 1983). For recordings of vagal motoneurons, prior to slicing, the brainstem–spinal cord was transferred to a 5 ml Sylgard-coated Petri dish that was constantly perfused with oxygenated aCSF. The brainstem–spinal cord was then pinned ventral side up. The 4th branch (rootlet) of the vagus nerve was isolated, as it is composed largely of axons that control glottal muscles that gate airflow into the lungs during ventilation in amphibians (Stuesse et al., 1984), and suctioned into a fine pipette containing 10% tetramethylrhodamine dextran (Invitrogen, Carlsbad, CA, USA). The dye was left in contact with the nerve root for at least 2 h to backfill the vagal motoneuron cell bodies, after which the tissue was sliced as previously described. All electrophysiological experiments described below were performed at room temperature (22°C).

### Electrophysiological protocols

#### Current-clamp recordings

**Fig. 1.**
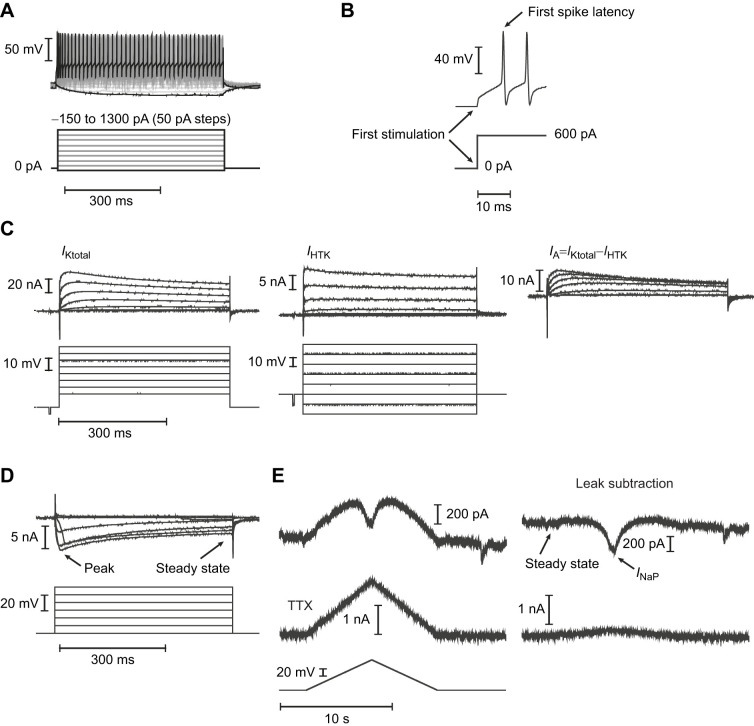
**Original traces from current-clamp and voltage-clamp experiments with motoneurons from control and overwintered American bullfrogs (*Aquarana catesbeiana*).** (A) A current-clamp experiment showing firing rates at injected currents spanning from −150 to 1300 pA (traces representing the responses to minimum and maximum input currents are highlighted with black lines) in incremental steps of 50 pA. (B) Details of the first spike latency after the first stimulation at 600 pA. (C) Original traces from currents recorded during voltage-clamp experiments. Inactivating potassium currents (*I*_A_) were obtained by subtracting high-threshold potassium currents (*I*_HTK_) from total potassium currents (*I*_Ktotal_). (D) Original traces from Ca^2+^ currents recorded during voltage-clamp experiments indicating the peak current and after it reached steady state. (E) Persistent Na^+^ inward currents (*I*_NaP_) were obtained after subtracting leak currents from the original recording. Tetrodotoxin (TTX; 500 nmol l^−1^) blocked the Na^+^ channel and abolished the *I*_NaP_.

The equipment used for whole-cell current-clamp experiments was identical to that of our previous studies (Zubov et al., 2022). Glass pipettes (thin walled) were fabricated using a Sutter Instruments puller (model P87, Novato, CA, USA) and had a resistance of ∼3 MΩ. After the whole-cell configuration had been obtained, we assessed electrophysiological properties in current clamp. We performed step protocols, where neurons were injected with −150 pA of current for 500 ms and then injected with +50 pA in incremental steps until 1300 pA, each step lasting 500 ms (Fig. 1A). The first spike latency was calculated from the moment of first stimulation until the first peak response at 600 pA for the hypoglossal and fast vagus motoneurons, and 200 pA for the slow vagus motoneurons (Fig. 1B). Rheobase was the first injected current eliciting an action potential.

#### Isolation of distinct K^+^ currents

Voltage-gated K^+^ currents were measured as previously described using standard aCSF with 500 nmol l^−1^ tetrodotoxin (TTX) and pipette filling solution (in mmol l^−1^: 110 potassium gluconate, 2 MgCl_2_, 10 Hepes, 1 Na_2_-ATP, 0.1 Na_2_-GTP and 2.5 EGTA; pH corrected to 7.2 with KOH; Gray and Santin, 2023). Briefly, tips of glass pipettes with a resistance of ∼2 MΩ were wrapped with Parafilm to reduce pipette capacitance. Upon break-in to the whole-cell mode, series resistance and whole-capacitance were compensated for by 85% using the amplifier circuitry. Because of the large size of these K^+^ currents, only cells with a stable series resistance of less than 12 MΩ uncompensated were included in the analysis, as these yield the most accurate assessments of large K^+^ currents with patch clamp (Gray and Santin, 2023). We then performed a two-step protocol to isolate K^+^ currents with distinct voltage sensitivities. To obtain the total outward K^+^ (*I*_Ktotal_), neurons were voltage clamped at −80 mV and then moved from −60 to +10 mV in 10 mV steps that lasted 500 ms. Next, we performed a protocol to isolate the high-threshold delayed rectifier K^+^ current (*I*_HTK_). The neuron was voltage clamped at −40 mV to inactivate depolarization-sensitive inactivating currents, and the step protocol was rerun as done for the total current. The difference between the currents produced in the protocol for *I*_Ktotal_ and *I*_HTK_ yielded the inactivating portion of the K^+^ current, consistent with the A-type K^+^ current (*I*_A_; Fig. 1C). These K^+^ currents are large, and we are aware of the voltage errors associated with measuring currents, especially large ones, in the whole-cell configuration (Gray and Santin, 2023). Yet, when we previously assessed these errors directly using a second voltage-following electrode, we found that large currents (∼20–30 nA) produced a voltage error of <10 mV at the 0 mV step when series resistance was kept below 12 MΩ and using the amplifier circuitry to compensate by 85%. Given the advantages of whole-cell patch clamp over sharp electrode recording, previous data suggest that relative comparisons across groups are valid for determining the effects of overwintering (i.e. increases or decreases across groups) (Gray and Santin, 2023; Li et al., 2004).

#### Measurement of L-type Ca^2+^ currents

Similar procedures were used to measure L-type Ca^2+^ currents (series resistance compensation). To isolate these currents from K^+^ and Na^+^ currents, we used a modified aCSF (in mmol l^−1^: 119 NaCl, 4 KCl, 1.4 MgCl_2_, 7.5 d-glucose, 10 Hepes, 20 TEA, 0.5 TTX, 2.5 CaCl_2_, bubbled with 98.5% O_2_, 1.5% CO_2_; pH 7.85) and parafilm-wrapped pipettes (2.5-4 MΩ) that were filled with solution containing (in mmol l^−1^): 95 CsCl, 2 MgCl_2_, 10 Hepes, 1 Na_2_-ATP, 0.1 Na_2_-GTP, 10 EGTA, 1 CaCl_2_ and 10 TEA-Cl (pH 7.2 corrected with CsOH). These solutions have been used in our previous study to isolate L-type Ca^2+^ currents by using internal and external TEA and Cs to block most of the K^+^ currents and TTX to block Na^+^ currents, leaving behind the Ca^2+^ currents (Bueschke et al., 2021b). The step protocol to activate the L-type Ca^2+^ current was as follows. Neurons were voltage clamped at −50 mV and then stepped up to +10 mV in 10 mV increments (0.5 s per step; Fig. 1D). The resulting inward current is consistent with the L-type Ca^2+^ current, which is partially inhibited by nimodipine (Bueschke et al., 2021b).

#### Persistent Na^+^ inward currents

We measured the persistent Na^+^ inward current (*I*_NaP_) utilizing a modified aCSF solution to block K^+^ and Ca^2+^ channels (in mmol l^−1^: 119 NaCl, 4 KCl, 1.4 MgCl_2_, 7.5 d-glucose, 10 Hepes, 20 TEA, 0.5 CdCl_2_ and 2.5 CaCl_2_; pH 7.85 corrected with NaOH). Pipettes (2.5–4.5 MΩ) were also wrapped with a Parafilm strip to reduce pipette capacitance and were filled with the same cesium-based solution used to record calcium currents described above. Currents were recorded during a protocol where cells were voltage clamped in a slow ramp (starting at −90 mV for 2 s, then ramp up to −20 mV for 5.8 s, then ramp back down to −90 mV for 5.8 s and keeping this voltage for 5 s; Fig. 1E). This protocol was repeated three times and values were averaged for analysis. Recordings in the presence of TTX confirmed blockage of the Na^+^ channels (Fig. 1E).

### Data analysis

For electrophysiological properties measured in current clamp, membrane potential was recorded as the voltage upon entry into the whole-cell configuration. Firing rate during current injection experiments was reported as the firing rate at each step. Input resistance was determined using Ohm's law (*R*_in_=*I*/*V*), dividing the voltage change elicited by the −150 pA step. If neurons were not at −60 mV, a bias current was applied to ensure the curves were obtained from same voltage. The total K^+^ current (*I*_Ktotal_) was measured at steady state at the end of the voltage step. The delayed rectifier current, *I*_HTK_, was also taken as the current at steady state. *I*_A_ was measured as the peak transient current. For the L-type Ca^2+^ current, we measured both peak and steady-state current. The peak Na^+^ current was calculated as the amplitude between the holding current at steady state and the peak response after leak subtraction of the original recording (Fig. 1E). The corresponding peak voltage was also recorded at the peak current response. Finally, the sensitivity of the current to voltage changes was calculated from the slope of the current response as Δ*I*/Δ*V* using the maximum voltage variation. Because of variability in cell size, all currents were normalized by whole-cell capacitance as measured on the amplifier during series resistance compensation, as cell capacitance is proportional to membrane surface area (an electrophysiology proxy for cell size).

Vagal motor neurons can be classified into two categories based on their intrinsic membrane properties: fast and slow firing (Zubov et al., 2021). Fast firing cells have lower membrane resistance and high maximal firing rates (typically <150 MΩ and >100 Hz), while slow firing cells have higher membrane resistance and low maximal firing rates (>200 MΩ and <50 Hz, entering depolarizing block at higher levels of current). For current-clamp experiments, we classified vagal motoneurons based on firing rates in combination with the presence/absence of depolarization block. For voltage-clamp experiments, we based this categorization on input resistance alone, as firing rate was not determined. In current-clamp experiments where we assessed both firing rate and input resistance, only 7 fast cells had *R*_in_ values above 200 MΩ (9.4% of all fast cells recorded in current clamp) and 5 slow cells had *R*_in_ values below 200 MΩ (9.8% of all slow cells recorded). Thus, while there is likely to be a small fraction of the cells miscategorized, this error is likely to be small and unlikely to influence interpretations in the data.

### Statistics

The effects of acclimation (control or overwintered) and injected current on firing rates, as well as the effects of acclimation and voltage on the ion current, were tested with a robust two-way ANOVA (Mair and Wilcox, 2020), followed by a false discovery rate *post hoc* test. Pairwise comparisons testing the effects of acclimation alone (control or overwintered) were made with Student’s *t*-test or a Wilcoxon test. Homogeneity of variances was tested with the Levene test, and normality with the Shapiro–Wilk test. Statistical differences were identified when *P*<0.05. Statistics were performed with R v.4.4.1 (https://posit.co/; http://www.R-project.org/; Wickham, 2016). All data are presented as means±s.d.

## RESULTS

### Excitability, passive membrane properties, firing latency and rheobase

**Fig. 2.**
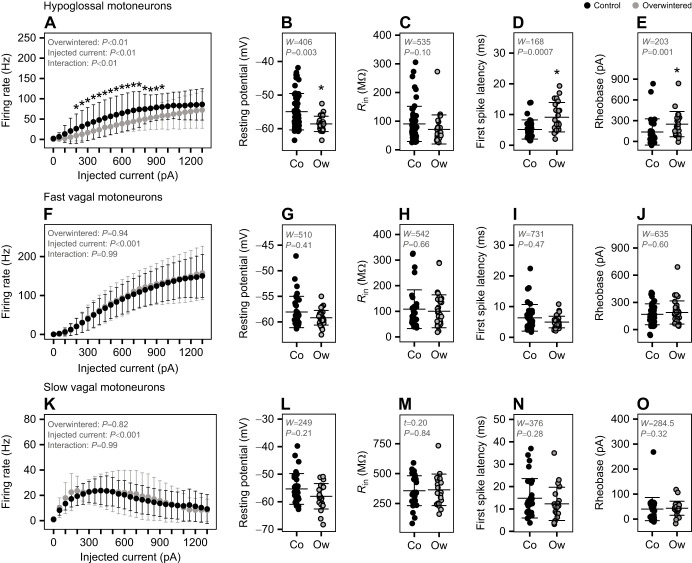
**Excitability, passive membrane properties, firing latency and rheobase in motoneurons from control and overwintered American bullfrogs.** Firing rate, resting membrane potential, input resistance (*R*_in_), first spike latency and rheobase in hypoglossal (A,B,C,D,E, respectively; *n*=33 control from 7 frogs; *n*=24 overwintered from 6 frogs), fast vagal (F,G,H,I,J, respectively; *n*=43 control from 9 frogs; *n*=19 overwintered from 9 frogs) and slow vagal (K,L,M,N,O, respectively; *n*=11 control from 9 frogs; *n*=12 overwintered from 9 frogs) motoneurons from control (Co) and overwintered (Ow) frogs. In A, F and K, the graphs show the statistical results for the robust two-way ANOVA followed by a false discovery rate *post hoc* test. In the remaining graphs, two group comparisons between control and overwintered bullfrogs were performed with a Wilcoxon test (*W*) or Student’s *t*-test (*P*). The asterisks indicate a statistically significant difference between control and overwintered bullfrogs (*P<0.05). Data are means±s.d.

To test the hypothesis that inactivity induces compensatory increases in the intrinsic excitability of motoneurons, we first assessed motoneuron firing rates in response to current injection in current clamp. Hypoglossal motoneurons from control animals displayed faster firing rates compared with overwintered cells with injected currents from 200 to 950 pA (Fig. 2A). In addition, the resting membrane potential of hypoglossal motoneurons from overwintered frogs was hyperpolarized relative to that of controls, while input resistance did not change (Fig. 2B,C). Consistent with these results, the time between stimulation and spike response (i.e. the first spike latency) was increased in hypoglossal motoneurons after overwintering (Fig. 2D), as was the minimum current necessary to elicit a spike (i.e. rheobase; Fig. 2E). In contrast, firing rate, membrane properties, first spike latency and rheobase did not change following overwintering in either fast or slow vagal motoneuron groups (Fig. 2F–O). Therefore, instead of compensation that enhances excitability, hypoglossal motoneurons reduced firing rates, while vagal motoneurons of both types did not change.

### K^+^ currents

**Fig. 3.**
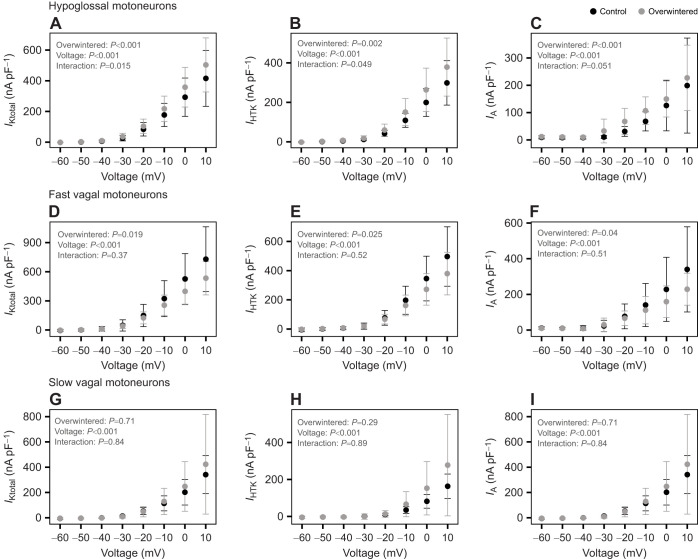
**K^+^ currents in motoneurons from control and overwintered American bullfrogs as a response to different voltages.** K^+^ currents were measured in the hypoglossal (A, *I*_Ktotal_; B, *I*_HTK_; C, *I*_A_; *n*=33 control from 7 frogs; *n*=24 overwintered from 8 frogs), fast vagal (D, *I*_Ktotal_; E, *I*_HTK_; F, *I*_A_; *n*=43 control from 9 frogs; *n*=19 overwintered from 7 frogs) and slow vagal (G, *I*_Ktotal_; H, *I*_HTK_; I, *I*_A_; *n*=11 control from 9 frogs; *n*=12 overwintered from 7 frogs) motoneurons. Differences were tested with a robust two-way ANOVA. Data are means±s.d.

To investigate specific mechanisms underlying plasticity of intrinsic excitability, we measured three voltage-dependent K^+^ currents that were previously identified in *A. catesbeiana* motoneurons (Gray and Santin, 2023). The total outward current (*I*_Ktotal_) was evoked during a step protocol in voltage clamp from a more negative holding voltage of −80 mV. Immediately after this protocol, the holding potential was changed to a more positive value (−50 mV) to inactivate the inactivating currents. We then ran the same step protocol again from this more positive holding potential to measure the non-inactivating delayed rectifier (*I*_HTK_; high-threshold K^+^) component. The difference between *I*_Ktotal_ and *I*_HTK_ reflects the K^+^ current that inactivates in a voltage-dependent manner, consistent with the A-type K^+^ current (*I*_A_). K^+^ current density of all K^+^ currents was increased in hypoglossal motoneurons following overwintering. The interaction effect identified for *I*_Ktotal_ and *I*_HTK_ indicates that the overwintered frogs displayed larger currents in response to voltage changes than the control group (Fig. 3A,B). Overwintering also increased the *I*_A_ in the hypoglossal motoneurons (Fig. 3C). Fast vagal motoneurons tended to display the highest current densities of all cell types measured, and the overwintering treatment elicited an overall group effect, where all K^+^ currents decreased after this treatment (Fig. 3D–F). K^+^ currents from slow vagal motoneurons were unaffected by overwintering (Fig. 3G–I). In sum, hypoglossal motoneurons enhanced the function of multiple K^+^ currents, consistent with reduced excitability, whereas fast vagal motoneurons decreased the K^+^ current function, and slow vagal motoneurons showed no change in K^+^ currents.

### L-Type Ca^2+^ currents

**Fig. 4.**
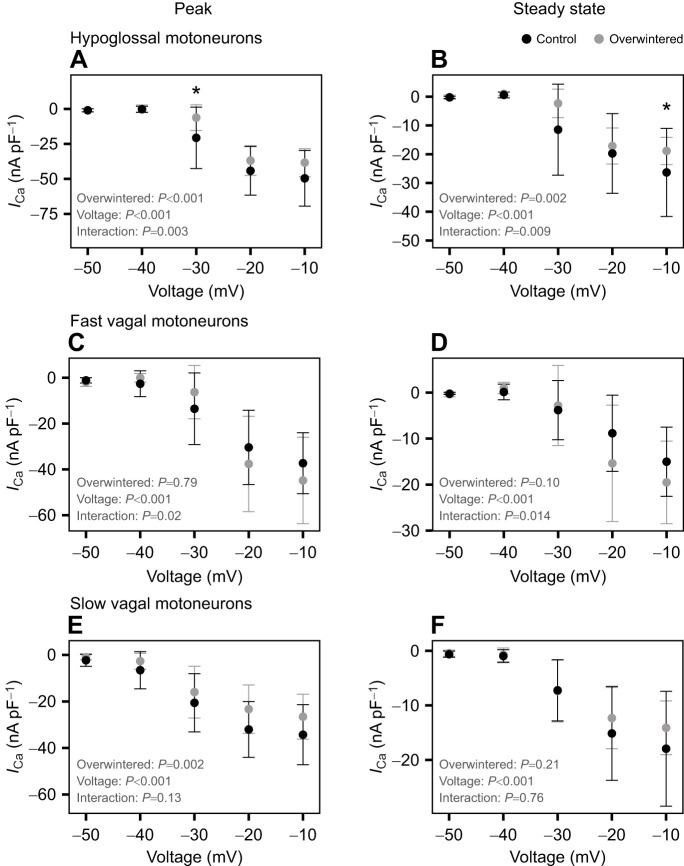
**Ca^2+^ currents in motoneurons from control and overwintered American bullfrogs as a response to different voltages.** Ca^2+^ currents (*I*_Ca_) were measured in the hypoglossal (A,B; *n*=25 control from 4 frogs; *n*=22 overwintered from 4 frogs), fast vagal (C,D; *n*=29 control from 8 frogs; *n*=27 overwintered from 6 frogs) and slow vagal (E,F; *n*=16 control from 8 frogs; *n*=17 overwintered from 6 frogs) motoneurons. The panels from each column represent the same type of current: peak: A,C,E; steady state: B,D,F. Differences were tested with a robust two-way ANOVA followed by a false discovery rate *post hoc* test. The asterisks indicate a statistically significant difference between motoneurons from control and overwintered bullfrogs (*P<0.05). Data are means±s.d.

L-Type Ca^2+^ currents play an important role in neuronal excitability through the regulation of burst firing. To investigate the plasticity of L-type Ca^2+^ currents, we recorded these currents in hypoglossal and vagal motoneurons following overwintering. The Ca^2+^ currents were isolated with TTX (to block *I*_Na_) and TEA (to block *I*_K_) and manifested as an inward current with slow inactivation characteristic of the L-type current. In hypoglossal motoneurons, both peak and steady-state Ca^2+^ current density decreased following overwintering, and the interaction between acclimation and voltage indicated a specific difference between treatments at −30 mV in peak current, and at −10 mV in steady-state current (Fig. 4A,B). In fast vagal motoneurons, although the interaction between voltage and treatment (control or overwintered frogs) was statistically significant in both peak and steady-state conditions, there was no group effect and the pairwise comparisons failed to detect any differences of Ca^2+^ currents between treatments (Fig. 4C,D). In contrast, slow vagal motoneurons had a reduced peak Ca^2+^ current in cold-acclimated animals, with no difference in the steady-state current (Fig. 4E,F). In sum, hypoglossal and slow vagal motoneurons have reduced Ca^2+^ currents, yet only hypoglossal motoneurons have decreased neuronal excitability. In contrast, fast vagal motoneurons have no change in either Ca^2+^ currents or neuronal excitability.

### Persistent Na^+^ inward currents

**Fig. 5.**
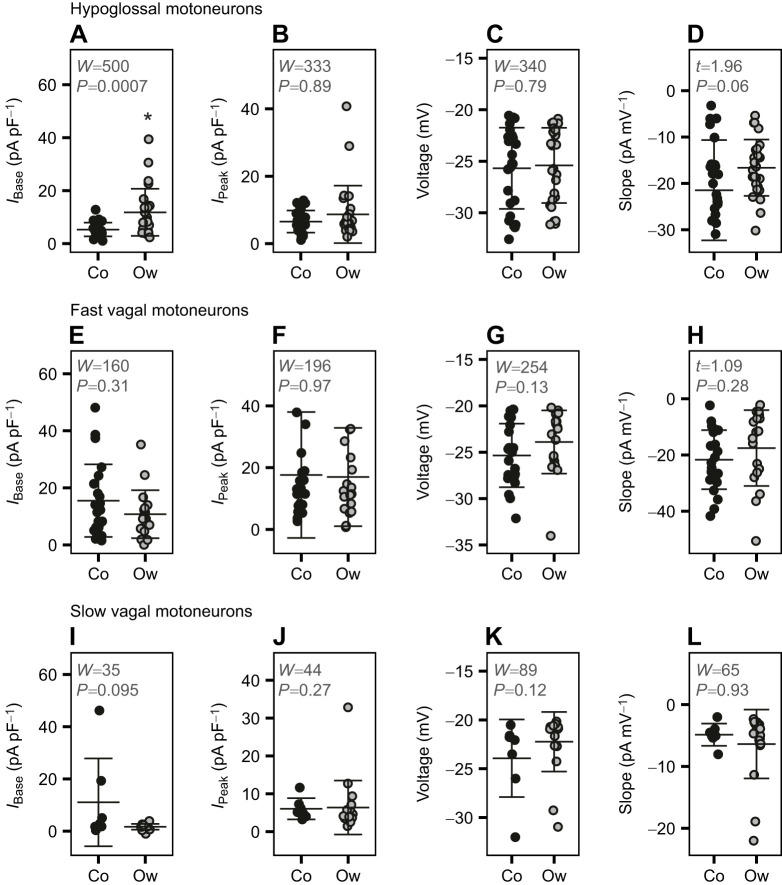
**Persistent Na^+^ inward currents (*I*_NaP_) in motoneurons from control and overwintered American bullfrogs as a response to voltage change.** Na^+^ holding current (*I*_Base_), peak current (*I*_Peak_), peak voltage and the sensitivity of the current to voltage change (slope) for hypoglossal (A,B,C,D, respectively; *n*=25 control from 7 frogs; *n*=26 overwintered from 6 frogs), fast vagal (E,F,G,H, respectively; *n*=22 control from 8 frogs; *n*=18 overwintered from 11 frogs) and slow vagal (I,J,K,L, respectively; *n*=7 control from 5 frogs; *n*=18 overwintered from 8 frogs) motoneurons. Differences between control and overwintered bullfrogs were tested with Wilcox or Student’s *t*-test. Data are means±s.d.

Persistent inward currents amplify and prolong synaptic input (Heckman et al., 2008; Lee and Heckman, 1996). Therefore, we hypothesized that plasticity of the persistent Na^+^ current could play a role in the compensatory response to hibernation. To investigate plasticity of the persistent Na^+^ inward current, we measured the holding current (*I*_Base_) and amplitude (*I*_Peak_) together with the voltage that induces the peak current response, as well as the sensitivity to voltage change in each cell type from control and overwintered frogs. In hypoglossal motoneurons, the holding Na^+^ current increased following overwintering compared with control cells (Fig. 5A), whereas peak current, peak voltage and the sensitivity of the current to voltage change were unaffected by overwintering (Fig. 5B–D). In vagal motoneurons, neither fast (Fig. 5E–H) nor slow cells (Fig. 5I–L) responded to overwintering for any of the measured variables. Slow vagus cells displayed similar currents and peak voltages to those of hypoglossal and fast vagal cells (Fig. 5I–K) but lower voltage sensitivity (Fig. 5D,H,L), as expected from high input resistance cells.

## DISCUSSION

Recent work on excitatory and inhibitory synapses indicates that the respiratory network uses plasticity that promotes motor performance after months of inactivity. Specifically, excitatory synaptic strength onto vagal motoneurons increases, while synaptic inhibition decreases in a way that promotes activity at cold temperatures (Amaral-Silva and Santin, 2023; Bueschke et al., 2021b, 2024; Saunders and Santin, 2024; Zubov et al., 2022). Therefore, we hypothesized that similar activity-dependent processes would alter the profiles of voltage-gated ion channels in the hypoglossal and vagus neurons to enhance excitability. Surprisingly, our results reveal that the most prevalent changes in motoneuron excitability stem from adjustments in ion channels that instead reduce or do not alter excitability.

### Methodological considerations

We performed these experiments using 1 month of cold acclimation to match our previous studies in this model (Amaral-Silva and Santin, 2023; Bueschke et al., 2021a, 2024; Zubov et al., 2022). In these cases, this acclimation period was sufficient to observe alterations at NMDA receptor and AMPA receptor synapses, as well as improvements in function during hypoxic conditions. While we show here that 1 month of simulating overwintering alters multiple ionic currents in different motoneuron types and reduced excitability of hypoglossal motoneurons, we cannot rule out the possibility that the results may differ with longer periods of acclimation. Therefore, carefully controlled studies varying the duration and pattern of simulated overwintering would be important to build upon these findings. In addition, animals used in this study, as well as our past studies, were raised in climate-controlled conditions and had never experienced a winter as adults until our experiments. It would be interesting in the future to address how developmental exposure to overwintering conditions or repeated overwintering events influences the neuronal properties we observed. Finally, ionic currents are generated by ion channel conductances and the electrochemical drive force, which is determined by the membrane potential and the Nernst potentials of the permeant ions. In this study, we kept ion concentrations fixed across all comparisons, which serves to best assess changes in conductance of ion channel activity between groups. However, if hibernation alters the concentration of intracellular or extracellular ions, this will alter ionic flux through the channels *in vivo*.

### Changes in motoneuron function after overwintering

The clearest neurophysiological adjustment we observed was that firing rates of hypoglossal motoneurons decreased after overwintering. This could be explained by a more negative resting membrane potential in addition to increases in multiple K^+^ currents and decreases in Ca^2+^ currents that are consistent with reduced firing rates. Mechanistically, because of its larger amplitude, high-threshold currents (*I*_HTK­_) play a dominant role in defining the total K^+^ current (*I*_Ktotal_) waveform, which in turn governs membrane repolarization during the action potential (Bouskila and Dudek, 1995). Increases in these currents likely contribute to reduced excitability by enhancing the relative refractory period, making fast repetitive firing more difficult (Exintaris and Lang, 1999; Humphries and Dart, 2015; Newkirk et al., 2022). Furthermore, firing rates can be governed by A-currents (*I*_A_) because of their lower threshold of activation (Amberg et al., 2003; Bouskila and Dudek, 1995). Therefore, the greater *I*_A_ could play a role in reduced excitability. Accordingly, the *I*_A_ also undergoes depolarization-dependent inactivation. Therefore, a more hyperpolarized resting membrane potential likely enhances the pool of channels that could be activated by reducing the fraction of inactivated channels at rest (Zhang et al., 2015), consequently enhancing *I*_A_. The lower hypoglossal excitability may also be connected with the reduced L-type Ca^2+^ current, which affects the depolarizing drive during the plateau potential, such that pharmacological reduction of L-type Ca^2+^ currents depresses motoneuron bursting (Harris-Warrick, 2002). In sum, hypoglossal motoneurons alter the ion channel profile in a way that is consistent with reduced excitability.

While our data provide mechanistic insight into the voltage-sensitive ionic mechanisms that reduce excitability of hypoglossal motoneurons, the mechanisms that account for hyperpolarization of the resting membrane potential are not clear at present. Given that we did not observe changes in input resistance at resting membrane potential, it is difficult to explain a more negative resting potential by opening or closing ion channels. One candidate that can alter the resting potential without altering input resistance is the Na^+^/K^+^-pump, as it carries a hyperpolarizing current when translocating three Na^+^ ions out of the cell for every two K^+^ ions in. While cold temperatures reduce Na^+^/K^+^-pump activity (Klee et al., 1974; Vassalle, 1987), it is possible that hypoglossal neurons upregulate the Na^+^/K^+^-pump leading to a more hyperpolarized membrane as we observed. In addition, increased Na^+^/K^+^-pump activity could also explain the larger holding current in hypoglossal motoneurons from cold-acclimated frogs (Fig. 5B). This rationale is corroborated by the fact that there were no differences in the input resistance for any of the cell types measured in this study.

Vagal motoneurons were separated into two types for analysis: fast firing with low input resistance and slow firing with higher input resistance (Zubov et al., 2021). In fast vagal motoneurons, overwintering was associated with maintenance of firing properties compared with controls. Interestingly, this maintenance of firing properties was associated with reduced K^+^ currents of all three types (Fig. 3), which opposes the expectation that a reduction of K^+^ currents would necessarily enhance excitability (Wulff et al., 2009). As such, similar firing rates in hibernators with downregulated voltage-gated K^+^ currents suggests that the function of other ion channels likely changed to maintain the output of these cells. Our data indicate that L-type Ca^2+^ and persistent Na^+^ currents are not likely candidates, as they did not change in fast vagal motoneurons. Likewise, we observed a similar trend for slow vagal motoneurons, where excitability was similar after overwintering but with a lowered density of L-type Ca^2+^ currents without any changes in the other currents measured. While we do not know the specific channels that play a compensatory role in maintaining vagal firing rates, it is now accepted that neurons may express dozens of ion channels with overlapping functions that can compensate and maintain electrophysiological properties (Goaillard and Marder, 2021). Although we measured most of the tractable broad classes of voltage-sensitive ionic currents expressed by these neurons, we were unable to voltage clamp the fast Na^+^ current or address the potentially large number of other channels that may influence excitability. In addition, we are unable to account for the interactions of individual currents on a cell-by-cell basis – such interactions are known to shape neuronal firing properties (Drion et al., 2015; Zhao and Golowasch, 2012). Regardless of the specific mechanisms at play, our results suggest that while vagal motoneurons did not alter their firing properties, similar firing properties after hibernation are likely to arise through different sets of ionic currents.

Overall, these results are at odds with the hypothesis that compensatory processes enhance excitability in response to inactivity associated with the hibernation environment as motoneurons either reduced or maintained excitability. Below, we present three non-mutually exclusive hypotheses for the potential functional implications of these results.

### Hypotheses for functional differentiation of motoneurons

#### Energy-savings hypothesis

If breathing must restart unabated after months of inactivity, why might hypoglossal motoneurons reduce their excitability? High-frequency firing in neurons is costly, presenting a large energy burden on neurons. Therefore, while neuronal excitability was indeed lower, we suggest that altering the profile of ion channels to achieve this goal might lower the ATP demands imposed by higher frequency firing, which could be adaptive upon emergence (Trevisiol et al., 2017). In addition, hypoglossal and slow vagal motoneurons exhibited reduced L-type Ca^2+^ currents. This lowering of Ca^2+^ permeability of the membrane mirrors previous work in vagal motoneurons, which has been suggested to be related to energy conservation (Bueschke et al., 2024). Specifically, we showed that a Ca^2+^-permeable glutamate receptor, the NMDA receptor, becomes less permeable to Ca^2+^ without altering its functional expression. The reason for this seems to be twofold. First, the Ca^2+^-pump is likely a costly mechanism because it hydrolyses one ATP for each Ca^2+^ ion transported. In comparison to Na^+^/K^+^-pumps, this corresponds to about three times the ATP used to transport Na^+^ and K^+^, as this pump transports three Na^+^ ions over a smaller electrochemical gradient. As the Ca^2+^-pump plays a major role in controlling Ca^2+^ homeostasis (Malci et al., 2022; Schmidt et al., 2017), reducing Ca^2+^ permeability could be a means of reducing the energetic costs of ion transport while neurons reallocate resources to other homeostatic mechanisms (Bueschke et al., 2024). Second, the augmented Ca^2+^ inflow through extra synaptic NMDA receptors may cause excitotoxicity (Szydlowska and Tymianski, 2010). Thus, the reduced Ca^2+^ permeability through L-type Ca^2+^ channels as well as the NMDA receptors could prevent excitotoxicity while optimizing energy allocation (Bueschke et al., 2024). In sum, multiple changes in ion channels may be associated with energy savings within individual motoneurons.

#### Neuron function hypothesis

We speculate that specific functions of the various motoneuron groups measured in the present study may play a role in the differential response we observed. The hypoglossal nerve innervates buccal cavity contractors alongside trigeminal, facial and vagus nerves. In amphibians, these muscles are responsible for elevation of the buccal floor, which forces air into the lungs when the glottis opens (Milsom et al., 2022). Unilateral sectioning of the bullfrog hypoglossal nerve reduces the peak buccal pressure by 28.8%, which is less than the contribution of the trigeminal nerve (43.3%; Sakakibara, 1984a,b). Therefore, while the hypoglossal nerve plays a role in the buccal force pump, it is not the main nerve governing buccal floor elevation in bullfrogs. In contrast, glottal opening is exclusively driven by the vagus nerve (Kogo et al., 1994; Milsom et al., 2022; Sakakibara, 1984a). Therefore, maintenance of the vagal motoneuron activity is essential for lung ventilation upon emergence from hibernation.

Regarding non-respiratory motor functions, the hypoglossal nerve is crucial in coordinating mouth gaping and tongue protrusion during feeding in amphibians (Deban et al., 2001; Nishikawa, 1999). As bullfrogs may recover from hibernation at temperatures as low as 12°C (Willis et al., 1956), it is possible that this may affect their feeding behavior. For instance, low temperatures may suppress appetite (Lillywhite et al., 1973) and reduce the rate of digestion in ectotherms, which could lead to regurgitation of the ingested food (Andrade et al., 2005). As such, a reduction in hypoglossal function would not be detrimental to survival upon emergence from hibernation as feeding would be suppressed by low environmental temperatures and the buccal pump could be maintained through compensatory increases in activity of other nerves. In contrast, as glottal opening is essential for respiration, vagal function must be maintained to survive emergence from hibernation when metabolic demands rapidly increase. Therefore, the function of motoneurons may dictate their responses to overwintering and how they perform upon emergence.

#### State dependence hypothesis

It is important to emphasize that all experiments in this study were performed under common conditions for experimental groups (oxygenated aCSF, 22°C, common ion concentrations, etc.). However, it was recently demonstrated that changes in glutamatergic and GABA systems may manifest as similar baseline activity patterns, with striking differences in output observed during stressors associated with emergence from the hibernation environment (Bueschke et al., 2024; Saunders and Santin, 2024). For example, NMDA-glutamate receptors become less permeable to Ca^2+^ and increase their degree of desensitization in response to repetitive stimulation. These synaptic adjustments do not affect baseline network activity under similar conditions used in this study, but instead promote stable activity in response to transient hypoxia likely to be encountered during emergence from hibernation (Bueschke et al., 2024). Furthermore, hibernators reduce GABAergic inhibition in the network. GABA signaling is essential for the expression of the respiratory rhythm in the adult anuran amphibian. Yet, baseline activity appears normal after hibernation despite a large reduction in GABA signaling. Beyond baseline activity, it was found that GABA normally plays a modulatory role to depress activity at cold temperatures; therefore, loss of GABA signaling in the respiratory network promotes activity at colder temperatures, likely aiding in restarting the respiratory network at cold temperatures during emergence from hibernation (Saunders and Santin, 2024). With these recent studies in mind, it is possible that the excitability profiles we identified in the present study may change under different environmental conditions. Accordingly, one intriguing hypothesis is that while hypoglossal motoneurons are less excitable and vagal motoneurons did not alter excitability at room temperature, perhaps changes in the ion channel profile are only unmasked at colder temperatures, similar to that which we observed for reduced GABAergic signaling.

### Perspectives for hibernators and inactivity

Most studies on the effects of dormancy on the neuromuscular system have focused on the nerve terminals at the neuromuscular junction (Bennett and Lavidis, 1991; Hudson et al., 2005; Lavidis et al., 2008; Vyskočil, 1976) and muscles. Conflicting results have been reported, where some did not identify differences in synaptic transmission between dormant and awake individuals (Bennett and Lavidis, 1991; Hudson et al., 2005), while others found significant differences (Vyskočil, 1976; Wernig et al., 1996), which have been attributed to species differences, ambient temperatures or dormancy duration (Hudson et al., 2005). Contrary to most studies, here we aimed to investigate explicitly the function of motoneuron physiology that controls orofacial behaviors. The present study revealed that different motoneurons contributing to the same behavior (i.e. respiration) respond differently to inactivity associated with overwintering. This adds a significant mechanistic variable that could be explored in future studies to understand the variations that drive motor behavior following dormancy in diverse organisms.

## References

[JEB249687C1] Amaral-Silva, L. d. and Santin, J. M. (2022). A brainstem preparation allowing simultaneous access to respiratory motor output and cellular properties of motoneurons in American bullfrogs. *J. Exp. Biol.* 225, jeb244079. 10.1242/jeb.244079 35574670 PMC9250796

[JEB249687C2] Amaral-Silva, L. and Santin, J. M. (2023). Synaptic modifications transform neural networks to function without oxygen. *BMC Biol.* 21, 54. 10.1186/s12915-023-01518-0 36927477 PMC10022038

[JEB249687C3] Amaral-Silva, L. and Santin, J. M. (2024). Neural processing without O_2_ and glucose delivery: from the pond to the clinic. *Physiol* 39. 10.1152/physiol.00030.2023 PMC11573265 38624246

[JEB249687C4] Amberg, G. C., Koh, S. D., Imaizumi, Y., Ohya, S. and Sanders, K. M. (2003). A-type potassium currents in smooth muscle. *Am. J. Physiol. Cell Physiol.* 284, C583-C595. 10.1152/ajpcell.00301.2002 12556357

[JEB249687C5] Andrade, D. V., Cruz-Neto, A. P., Abe, A. S. and Wang, T. (2005). Specific dynamic action in ectothermic vertebrates: A review of the determinants of postprandial metabolic response in fishes, amphibians, and reptiles. In *Physiological and Ecological Adaptations to Feeding in Vertebrates* (ed. J. M. Starck and T. Wang), pp. 306-324. Enfield, NH: Science Publishers.

[JEB249687C6] Bennett, M. R. and Lavidis, N. A. (1991). Probabilistic secretion of quanta from the release sites of nerve terminals in amphibian muscle modulated by seasonal changes. *Neurosci. Lett.* 134, 79-82. 10.1016/0304-3940(91)90513-S 1687704

[JEB249687C7] Bonaldo, P. and Sandri, M. (2013). Cellular and molecular mechanisms of muscle atrophy. *Dis. Model. Mech.* 6, 25-39. 10.1242/dmm.010389 23268536 PMC3529336

[JEB249687C8] Bouskila, Y. and Dudek, F. E. (1995). A rapidly activating type of outward rectifier K^+^ current and A–current in rat suprachiasmatic nucleus neurones. *J. Physiol.* 488, 339-350. 10.1113/jphysiol.1995.sp020970 8568674 PMC1156674

[JEB249687C9] Bueschke, N., Amaral-Silva, L. d., Adams, S. and Santin, J. M. (2021a). Transforming a neural circuit to function without oxygen and glucose delivery. *Curr. Biol.* 31, R1564-R1565. 10.1016/j.cub.2021.11.003 34932961 PMC8711626

[JEB249687C10] Bueschke, N., Amaral-Silva, L., Hu, M. and Santin, J. M. (2021b). Lactate ions induce synaptic plasticity to enhance output from the central respiratory network. *J. Physiol.* 599, 5485-5504. 10.1113/JP282062 34761806 PMC8696744

[JEB249687C11] Bueschke, N., Amaral-Silva, L., Hu, M., Alvarez, A. and Santin, J. M. (2024). Plasticity in the functional properties of NMDA receptors improves network stability during severe energy stress. *J. Neurosci.* 44, e0502232024. 10.1523/JNEUROSCI.0502-23.2024 38262722 PMC10903970

[JEB249687C12] Clark, B. C., Manini, T. M., Bolanowski, S. J. and Ploutz-Snyder, L. L. (2006). Adaptations in human neuromuscular function following prolonged unweighting: II. Neurological properties and motor imagery efficacy. *J. Appl. Physiol.* 101, 264-272. 10.1152/japplphysiol.01404.2005 16514003

[JEB249687C13] Cormery, B., Beaumont, E., Csukly, K. and Gardiner, P. (2005). Hindlimb unweighting for 2 weeks alters physiological properties of rat hindlimb motoneurones. *J. Physiol.* 568, 841-850. 10.1113/jphysiol.2005.091835 16123107 PMC1464183

[JEB249687C14] Cotton, C. J. (2016). Skeletal muscle mass and composition during mammalian hibernation. *J. Exp. Biol.* 219, 226-234. 10.1242/jeb.125401 26792334

[JEB249687C15] Deban, S. M., O'Reilly, J. C. and Nishikawa, K. C. (2001). The evolution of the motor control of feeding in amphibians. *Am. Zool.* 41, 1280-1298.

[JEB249687C16] Desai, N. S., Rutherford, L. C. and Turrigiano, G. G. (1999). Plasticity in the intrinsic excitability of cortical pyramidal neurons. *Nat. Neurosci.* 2, 515-520. 10.1038/9165 10448215

[JEB249687C17] Deschenes, M. R., Giles, J. A., McCoy, R. W., Volek, J. S., Gomez, A. L. and Kraemer, W. J. (2002). Neural factors account for strength decrements observed after short-term muscle unloading. *Am. J. Physiol. Regul. Integr. Comp. Physiol.* 282, R578-R583. 10.1152/ajpregu.00386.2001 11792669

[JEB249687C18] Dobson, G. P. (2004). Organ arrest, protection and preservation: natural hibernation to cardiac surgery. *Comp. Biochem. Physiol. B* 139, 469-485. 10.1016/j.cbpc.2004.06.002 15544969

[JEB249687C19] Drion, G., O'Leary, T. and Marder, E. (2015). Ion channel degeneracy enables robust and tunable neuronal firing rates. *Proc. Natl. Acad. Sci. USA* 112, E5361-70. 10.1073/pnas.1516400112 26354124 PMC4586887

[JEB249687C20] Exintaris, B. and Lang, R. J. (1999). K^+^ channel blocker modulation of the refractory period in spontaneously active guinea-pig ureters. *Urol. Res.* 27, 319-327. 10550519 10.1007/pl00006605

[JEB249687C21] Goaillard, J.-M. and Marder, E. (2021). Ion channel degeneracy, variability, and covariation in neuron and circuit resilience. *Annu. Rev. Neurosci.* 44, 335-357. 10.1146/annurev-neuro-092920-121538 33770451

[JEB249687C22] Golowasch, J., Casey, M., Abbott, L. F. and Marder, E. (1999). Network stability from activity-dependent regulation of neuronal conductances. *Neural Comput.* 11, 1079-1096. 10.1162/089976699300016359 10418158

[JEB249687C23] Gray, M. and Santin, J. M. (2023). Series resistance errors in whole cell voltage clamp measured directly with dual patch-clamp recordings: not as bad as you think. *J. Neurophysiol.* 129, 1177-1190. 10.1152/jn.00476.2022 37073967 PMC10190937

[JEB249687C24] Harris-Warrick, R. M. (2002). Voltage-sensitive ion channels in rhythmic motor systems. *Curr. Opin. Neurobiol.* 12, 646-651. 10.1016/S0959-4388(02)00377-X 12490254

[JEB249687C25] Heckman, C. J., Johnson, M., Mottram, C. and Schuster, J. (2008). Persistent inward currents in spinal motoneurons and their influence on human motoneuron firing patterns. *Neuroscientist* 14, 264-275. 10.1177/1073858408314986 18381974 PMC3326417

[JEB249687C26] Hudson, N. J. and Franklin, C. E. (2002). Maintaining muscle mass during extended disuse: aestivating frogs as a model species. *J. Exp. Biol.* 205, 2297-2303. 10.1242/jeb.205.15.2297 12110663

[JEB249687C27] Hudson, N. J., Lavidis, N. A., Choy, P. T. and Franklin, C. E. (2005). Effect of prolonged inactivity on skeletal motor nerve terminals during aestivation in the burrowing frog, *Cyclorana alboguttata*. *J. Comp. Physiol. A* 191, 373-379. 10.1007/s00359-004-0593-5 15647924

[JEB249687C28] Humphries, E. S. A. and Dart, C. (2015). Neuronal and cardiovascular potassium channels as therapeutic drug targets: promise and pitfalls. *J. Biomol. Screen* 20, 1055-1073. 10.1177/1087057115601677 26303307 PMC4576507

[JEB249687C29] Ivakine, E. A. and Cohn, R. D. (2014). Maintaining skeletal muscle mass: lessons learned from hibernation. *Exp. Physiol.* 99, 632-637. 10.1113/expphysiol.2013.074344 24443348

[JEB249687C30] Klee, M. R., Pierau, F.-K. and Faber, D. S. (1974). Temperature effects on resting potential and spike parameters of cat motoneurons. *Exp. Brain Res.* 19, 478-492. 4854613 10.1007/BF00236112

[JEB249687C31] Kogo, N. and Remmers, J. E. (1994). Neural organization of the ventilatory activity in the frog, *Rana catesbeiana*. II. *J. Neurobiol.* 25, 1080-1094. 10.1002/neu.480250905 7815065

[JEB249687C32] Kogo, N., Perry, S. F. and Remmers, J. E. (1994). Neural organization of the ventilatory activity in the frog, *Rana catesbeiana*. I. *J. Neurobiol.* 25, 1067-1079. 10.1002/neu.480250904 7815064

[JEB249687C33] Lavidis, N. A., Hudson, N. J., Choy, P. T., Lehnert, S. A. and Franklin, C. E. (2008). Role of calcium and vesicle-docking proteins in remobilising dormant neuromuscular junctions in desert frogs. *J. Comp. Physiol. A* 194, 27-37. 10.1007/s00359-007-0284-0 17987295

[JEB249687C34] Lee, R. H. and Heckman, C. J. (1996). Influence of voltage-sensitive dendritic conductances on bistable firing and effective synaptic current in cat spinal motoneurons in vivo. *J. Neurophysiol.* 76, 2107-2110. 10.1152/jn.1996.76.3.2107 8890322

[JEB249687C35] LeMasson, G., Marder, E. and Abbott, L. F. (1993). Activity-dependent regulation of conductances in model neurons. *Science* 259, 1915-1917. 10.1126/science.8456317 8456317

[JEB249687C36] Li, M., West, J. W., Lai, Y., Scheuer, T. and Catterall, W. A. (1992). Functional modulation of brain sodium channels by cAMP-dependent phosphorylation. *Neuron* 8, 1151-1159. 10.1016/0896-6273(92)90135-Z 1319185

[JEB249687C37] Li, W.-C., Soffe, S. R. and Roberts, A. (2004). A direct comparison of whole cell patch and sharp electrodes by simultaneous recording from single spinal neurons in frog tadpoles. *J. Neurophysiol.* 92, 380-386. 10.1152/jn.01238.2003 14999043

[JEB249687C38] Lillywhite, H. B., Licht, P. and Chelgren, P. (1973). The role of behavioral thermoregulation in the growth energetics of the toad, *Bufo boreas*. *Ecology* 54, 375-383. 10.2307/1934345

[JEB249687C39] Mair, P. and Wilcox, R. (2020). Robust statistical methods in R using the WRS2 package. *Behav. Res. Methods* 52, 464-488. 10.3758/s13428-019-01246-w 31152384

[JEB249687C40] Malci, A., Lin, X., Sandoval, R., Gundelfinger, E. D., Naumann, M., Seidenbecher, C. I. and Herrera-Molina, R. (2022). Ca^2+^ signaling in postsynaptic neurons: neuroplastin-65 regulates the interplay between plasma membrane Ca^2+^ ATPases and ionotropic glutamate receptors. *Cell Calcium* 106, 102623. 10.1016/j.ceca.2022.102623 35853264

[JEB249687C41] Marder, E. and Prinz, A. A. (2002). Modeling stability in neuron and network function: the role of activity in homeostasis. *BioEssays* 24, 1145-1154. 10.1002/bies.10185 12447979

[JEB249687C42] Milsom, W. K., Gilmour, K. M., Perry, S., Gargaglioni, L. H., Hedrick, M. S., Kinkead, R. and Wang, T. (2022). Control of breathing in ectothermic vertebrates. In *Comprehensive Physiology* (ed. Y. S. Prakash), pp. 3869-3988. Wiley. 10.1002/cphy.c210041 35997081

[JEB249687C43] Neto, A. V. G. S., Filogonio, R. and Leite, C. A. C. (2024). Recovery of the baroreflex and autonomic modulation after anesthesia with MS-222 in bullfrogs. *Comp. Biochem. Physiol. A* 295, 111654. 10.1016/j.cbpa.2024.111654 38729257

[JEB249687C44] Newkirk, G. S., Guan, D., Dembrow, N., Armstrong, W. E., Foehring, R. C. and Spain, W. J. (2022). Kv2.1 potassium channels regulate repetitive burst firing in extratelencephalic neocortical pyramidal neurons. *Cereb. Cortex* 32, 1055-1076. 10.1093/cercor/bhab266 34435615 PMC8889945

[JEB249687C45] Nishikawa, K. C. (1999). Neuromuscular control of prey capture in frogs. *Phil. Trans. R. Soc. Lond. B* 354, 941-954. 10.1098/rstb.1999.0445 10382226 PMC1692590

[JEB249687C46] Ratigan, E. D. and McKay, D. B. (2016). Exploring principles of hibernation for organ preservation. *Transplant. Rev.* 30, 13-19. 10.1016/j.trre.2015.08.002 26613668

[JEB249687C47] Sakakibara, Y. (1984a). Trigeminal nerve activity and buccal pressure as an index of total inspiratory activity in the bullfrog. *Jpn. J. Physiol.* 34, 827-838. 10.2170/jjphysiol.34.827 6335902

[JEB249687C48] Sakakibara, Y. (1984b). The pattern of respiratory nerve activity in the bullfrog. *Jpn. J. Physiol.* 34, 269-282. 10.2170/jjphysiol.34.269 6332228

[JEB249687C49] Santin, J. M. (2019). Motor inactivity in hibernating frogs: Linking plasticity that stabilizes neuronal function to behavior in the natural environment. *Dev. Neurobiol.* 79, 880-891. 10.1002/dneu.22721 31584749

[JEB249687C50] Santin, J. M. and Hartzler, L. K. (2016). Control of lung ventilation following overwintering conditions in bullfrogs, *Lithobates catesbeianus*. *J. Exp. Biol.* 219, 2003-14. 10.1242/jeb.136259 27091862

[JEB249687C51] Santin, J. M. and Hartzler, L. K. (2017). Activation of respiratory muscles does not occur during cold-submergence in bullfrogs, *Lithobates catesbeianus*. *J. Exp. Biol.* 220, 1181-1186. 10.1242/jeb.153544 28096431

[JEB249687C52] Santin, J. M., Vallejo, M. and Hartzler, L. K. (2017). Synaptic up-scaling preserves motor circuit output after chronic, natural inactivity. *eLife* 6, e30005. 10.7554/eLife.30005 28914603 PMC5636609

[JEB249687C53] Saunders, S. E. and Santin, J. M. (2024). Hibernation reduces GABA signaling in the brainstem to enhance motor activity of breathing at cool temperatures. *BMC Biol.* 22, 251. 10.1186/s12915-024-02050-5 39497096 PMC11533357

[JEB249687C54] Schmidt, N., Kollewe, A., Constantin, C. E., Henrich, S., Ritzau-Jost, A., Bildl, W., Saalbach, A., Hallermann, S., Kulik, A., Fakler, B. et al. (2017). Neuroplastin and basigin are essential auxiliary subunits of plasma membrane Ca^2+^-ATPases and key regulators of Ca^2+^ clearance. *Neuron* 96, 827-838.e9. 10.1016/j.neuron.2017.09.038 29056295

[JEB249687C55] Seki, K., Kizuka, T. and Yamada, H. (2007). Reduction in maximal firing rate of motoneurons after 1-week immobilization of finger muscle in human subjects. *J. Electromyogr. Kinesiol.* 17, 113-120. 10.1016/j.jelekin.2005.10.008 16448820

[JEB249687C56] Shaw, G. (1802). *General Zoology or Systematic Natural History, Part 1*, Vol. III. Amphibia, London: Thomas Davison.

[JEB249687C57] Stuesse, S. L., Cruce, W. L. R. and Powell, K. S. (1983). Afferent and efferent components of the hypoglossal nerve in the grass frog, *Rana pipiens*. *J. Comp. Neurol.* 217, 432-439. 10.1002/cne.902170407 6604074

[JEB249687C58] Stuesse, S. L., Cruce, W. L. R. and Powell, K. S. (1984). Organization within the cranial IX–X complex in ranid frogs: a horseradish peroxidase transport study. *J. Comp. Neurol.* 222, 358-365. 10.1002/cne.902220304 6607937

[JEB249687C59] Szydlowska, K. and Tymianski, M. (2010). Calcium, ischemia and excitotoxicity. *Cell Calcium* 47, 122-129. 10.1016/j.ceca.2010.01.003 20167368

[JEB249687C60] Tattersall, G. J. and Ultsch, G. R. (2008). Physiological ecology of aquatic overwintering in ranid frogs. *Biol. Rev.* 83, 119-140. 10.1111/j.1469-185X.2008.00035.x 18429765

[JEB249687C61] Trevisiol, A., Saab, A. S., Winkler, U., Marx, G., Imamura, H., Möbius, W., Kusch, K., Nave, K.-A. and Hirrlinger, J. (2017). Monitoring ATP dynamics in electrically active white matter tracts. *eLife* 6, e24241. 10.7554/eLife.24241 28414271 PMC5415357

[JEB249687C62] Turrigiano, G. G. (2008). The self-tuning neuron: synaptic scaling of excitatory synapses. *Cell* 135, 422-435. 10.1016/j.cell.2008.10.008 18984155 PMC2834419

[JEB249687C63] Turrigiano, G. (2011). Too many cooks? Intrinsic and synaptic homeostatic mechanisms in cortical circuit refinement. *Annu. Rev. Neurosci.* 34, 89-103. 10.1146/annurev-neuro-060909-153238 21438687

[JEB249687C64] Turrigiano, G., Abbott, L. F. and Marder, E. (1994). Activity-dependent changes in the intrinsic properties of cultured neurons. *Science* 264, 974-977. 10.1126/science.8178157 8178157

[JEB249687C65] Vassalle, M. (1987). Contribution of the Na^+^/K^+^-pump to the membrane potential. *Experientia* 43, 1135-1140. 10.1007/BF01945511 2446906

[JEB249687C66] Vyskočil, F. (1976). Miniature end-plate potentials and sensitivity to acetylcholine in the fast and slow limb muscles of hibernating golden hamsters. *Pflügers Arch* 361, 165-167. 10.1007/BF00583461 943090

[JEB249687C67] Wen, W. and Turrigiano, G. G. (2024). Keeping your brain in balance: homeostatic regulation of network function. *Annu. Rev. Neurosci.* 47, 41-61. 10.1146/annurev-neuro-092523-110001 38382543

[JEB249687C68] Wernig, A., Dorlöchter, M. and Palazis, P. (1996). Differential sensitivity to Mg^2+^- and tubocurarine-block of frog neuromuscular junctions in summer and winter. *Neurosci. Lett.* 207, 41-44. 10.1016/0304-3940(96)12483-6 8710205

[JEB249687C69] Wickham, H. (2016). *ggplot2: Elegant Graphics for Data Analysis*. New York: Springer-Verlag.

[JEB249687C70] Wickler, S. J., Horwitz, B. A. and S.Kott, K. (1987). Muscle function in hibernating hamsters: A natural analog to bed rest? *J. Therm. Biol.* 12, 163-166. 10.1016/0306-4565(87)90058-1

[JEB249687C71] Wickler, S. J., Hoyt, D. F. and Van Breukelen, F. (1991). Disuse atrophy in the hibernating golden-mantled ground squirrel, *Spermophilus lateralis*. *Am. J. Physiol. Regul. Integr. Comp. Physiol.* 261, R1214-R1217. 10.1152/ajpregu.1991.261.5.R1214 1951770

[JEB249687C72] Willis, Y. L., Moyle, D. L. and Baskett, T. S. (1956). Emergence, breeding, hibernation, movements and transformation of the bullfrog, *Rana catesbeiana*, in Missouri. *Copeia* 1956, 30-41. 10.2307/1439241

[JEB249687C73] Withers, P. C. and Cooper, C. E. (2006). Metabolic depression: a historical perspective. In *Aestivation: Molecular and Physiological Aspects* (ed. Carlos Arturo Navas and José Eduardo Carvalho), pp. 1-23. Heidelberg: Springer Berlin.

[JEB249687C74] Wulff, H., Castle, N. A. and Pardo, L. A. (2009). Voltage-gated potassium channels as therapeutic targets. *Nat. Rev. Drug. Discov.* 8, 982-1001. 10.1038/nrd2983 19949402 PMC2790170

[JEB249687C75] Yacoe, M. E. (1983). Maintenance of the pectoralis muscle during hibernation in the big brown bat, *Eptesicus fuscus*. *J. Comp. Physiol. B* 152, 97-104. 10.1007/BF00689733

[JEB249687C76] Zhang, H.-Y., Picton, L., Li, W.-C. and Sillar, K. T. (2015). Mechanisms underlying the activity-dependent regulation of locomotor network performance by the Na^+^ pump. *Sci. Rep.* 5, 16188. 10.1038/srep16188 26541477 PMC4635428

[JEB249687C77] Zhao, S. and Golowasch, J. (2012). Ionic current correlations underlie the global tuning of large numbers of neuronal activity attributes. *J. Neurosci.* 32, 13380-13388. 10.1523/JNEUROSCI.6500-11.2012 23015428 PMC3541048

[JEB249687C78] Zubov, T., Silika, S., Dukkipati, S. S., Hartzler, L. K. and Santin, J. M. (2021). Characterization of laryngeal motor neuron properties in the American bullfrog. *Lithobates catesbieanus*. *Resp. Physiol.* 294, 103745. 10.1016/j.resp.2021.103745 PMC8484031 34298168

[JEB249687C79] Zubov, T., Amaral-Silva, L. d. and Santin, J. M. (2022). Inactivity and Ca^2+^ signaling regulate synaptic compensation in motoneurons following hibernation in American bullfrogs. *Sci. Rep.* 12, 11610. 10.1038/s41598-022-15525-8 35803955 PMC9270477

